# Diagnostic Value of Interferon-γ Release Assays on Pericardial Effusion for Diagnosis of Tuberculous Pericarditis

**DOI:** 10.1371/journal.pone.0165008

**Published:** 2016-10-18

**Authors:** Sainan Bian, Yueqiu Zhang, Lifan Zhang, Xiaochun Shi, Xiaoqing Liu

**Affiliations:** 1 Department of Infectious Diseases, Peking Union Medical College Hospital, Chinese Academy of Medical Sciences & Peking Union Medical College, Beijing, China; 2 Clinical Epidemiology Unit, Peking Union Medical College, International Clinical Epidemiology Network, Beijing, China; Chinese Academy of Medical Sciences and Peking Union Medical College, CHINA

## Abstract

Diagnosis of tuberculous pericarditis remains a challenge. We aimed in this study to evaluate the diagnostic value of T-SPOT.*TB* on pericardial effusion for diagnosis of tuberculous pericarditis. Patients with suspected tuberculous pericarditis were enrolled consecutively between August 2011 and December 2015. T-SPOT.*TB* was performed on both pericardial effusion mononuclear cells (PEMCs)and peripheral blood mononuclear cells (PBMCs). Sensitivity, specificity, predictive value (PV), and likelihood ratio (LR) of T-SPOT.*TB* on PEMCs and PBMCs were analyzed. Among the 75 patients enrolled, 24 patients (32%) were diagnosed with tuberculous pericarditis, 38 patients (51%) with nontuberculous pericarditis, and 13 patients (17%) were clinically indeterminate and were excluded from the final analysis. The sensitivity, specificity, positive PV (PPV), negative PV (NPV), positive LR (LR+), and negative LR (LR-) of T-SPOT.*TB* on PEMCs was 92%,92%,88%,95%,11.61, and 0.09, respectively, compared to 83%, 95%, 91%, 90%,15.83, and 0.18, respectively of T-SPOT.*TB* on PBMCs. In patients with tuberculous pericarditis, the median frequencies of spot-forming cells (SFCs) of T-SPOT.*TB* on PEMCs and PBMCs was 172SFCs/10^6^MCs (IQR 39~486), and 66 SFCs/10^6^MCs (IQR 24~526), respectively, but the difference was not statistically significant (P = 0.183). T-SPOT.*TB* on PEMCs appeared to be a valuable and rapid diagnostic method for diagnosis of tuberculous pericarditis with high sensitivity and specificity.

## Introduction

*Tuberculosis* (*TB*) remains a health challenge in the world. 9.6 million people are estimated to have developed *TB* in 2014 worldwide, and China accounted for 10% of the total *TB* cases[1]. As a form of extrapulmonary tuberculosis, tuberculous pericarditis, is found in approximately 1% of *TB* cases in autopsy studies and in 1% to 2% of cases with pulmonary *TB*. It is the most common cause of pericarditis in countries with high *TB* burden [2]. Tuberculous pericarditis has a high mortality of 26% at 6 months, and early diagnosis and treatment are crucial [3]. However, its diagnosis remains a challenge since the sensitivity of the golden standard, microbiological examination, is low[2].

Interferon (IFN)-γ release assays (IGRAs), a new generation of *TB* diagnostic assays, have recently shown promising results in diagnosing active extrapulmonary *TB*[4]. Particularly, previous studies have shown that T-SPOT.*TB* on serous effusion and cerebral spinal fluid have a higher diagnostic accuracy for tuberculous serositis and tuberculous meningitis, compared to T-SPOT.*TB* on peripheral blood mononuclear cells(PBMCs)[5, 6]. However, the diagnostic value of T-SPOT.*TB* on pericardial effusion has been rarely reported. In this study, we sought to evaluate the diagnostic value of T-SPOT.*TB* on pericardial effusion for patients with tuberculous pericarditis.

## Materials and Methods

### Study participants

All patients with suspected tuberculous pericarditis were enrolled consecutively between August 1^st^, 2011 and December 31^st^, 2015 at Peking Union Medical College Hospital (PUMCH). Included patients had to be followed up for at least three months from discharge in order to see the effect of treatments. We conducted this study in April 2016. The exclusion criterion included patients without T-SPOT.*TB* on pericardial effusion or peripheral blood, indeterminate results of T-SPOT.*TB* and loss to follow up. We received a waiver of ethics approval from the Institutional Review Board at our institution because this was a retrospective and observational study. Patient information was anonymized and de-identified prior to analysis.

Clinical information was extracted from patients’ medical records retrospectively. The diagnosis was made based on clinical manifestations, radiology, microbiological results, histopathological results, and effect of anti-*TB* treatment. It was given by the research physicians when follow-up was completed, independent of either the T-SPOT.*TB* results or the clinical diagnosis given by the treating physician. If the two physicians had different opinions of the final diagnosis, a third researcher was referred. All patients were given HIV test. Pericardial effusion was obtained by pericardiocentesis or during the operation of fenestration pericardium or pericardiectomy. Other routine tests performed included routine cell counting, microscopy (Gram stain, acid-fast bacilli stain), *bacterial* culture, *fungal* culture, *TB* polymerase chain reaction (PCR) (Roche Amplicor), *Mycobacterium* culture (Liquid culture method, BD MGIT960), and colloidal gold method of Kabelykit to identify *Mycobacterium tuberculosis* (*MTB*). Heparinized samples of venous blood (4 ml) and of pericardial effusion (50 ml) were obtained and processed for detecting specific T cell responses to 1 region of difference(RD1) encoded antigens by T-SPOT.*TB*(Oxford Immunotec, Abingdon, UK).

### Diagnostic categories

Based on previous publications[5, 7], diagnostic categories are shown in Table 1.

**Table 1:** Diagnostic category of tuberculous pericarditis.

Diagnostic category	Criteria
**Confirmed *TB***	Identification of the bacillus in pericardial fluid or biopsy specimen by acid-fast bacilli stain, culture, and/or by polymerase chain reaction (PCR), or by the presence of granulomas in pericardial biopsy tissue Positive result of sputum acid-fast bacilli stain, culture, and/or by PCR in the presence of clinical and radiologic evidence of *TB* and in the absence of any other obvious cause associated with pericardial effusions
**Highly probable *TB***	Clinical and radiologic evidence of *TB* in the absence of any other obvious cause with a positive response to antituberculous therapy
**Non-*TB***	Effusion/sputum acid-fast bacilli stain and *TB*-culture-negative, an alternative diagnosis was established, improved without anti-*TB* treatment
**Clinically indeterminate**	Effusions of unknown origin (that is, all possible etiologic causes could not be excluded)

### T-SPOT.*TB* on PEMCs and PBMCs

50 ml of pericardial effusion and 4 ml of peripheral blood were collected from each patient and were performed within six hours after collection by laboratory staff blinded to patients’ clinical data. T-SPOT.*TB* utilized AIM-V (GIBCOTMAIM V Medium liquid, Invitrogen, US) as negative control, PHA as positive control, and early-secreted antigenic target 6-kDa protein(ESAT-6) and culture filtrate protein 10 (CFP-10) as specific antigens, respectively. Pericardial effusion mononuclear cells (PEMCs) were separated by Ficoil-Hypaque gradient centrifugation and plated (2.5×10^5^ per well) on a plate pre-coated with an antibody against interferon-γ. After incubation 16–18 h at 37°C in 5% carbon dioxide, plate wells were washed and incubated with a conjugate against the antibody used and an enzyme substrate. Spot-forming cells (SFCs) that represented antigen-specific T cells secreting interferon-γ were counted with an automated ELISPOT reader (AID-ispot, Strassberg, Germany). A positive response was defined as six or more spots and had twice the number of spots than the negative control well. The background number of spots in the negative control well for PBMCs and PEMCs should be less than ten and twenty spots, respectively [5].

### Statistical analysis

Sensitivity, specificity, positive predictive value (PPV), negative predictive value (NPV), positive likelihood ratio (LR+), and negative likelihood ratio (LR-) were calculated to evaluate the diagnostic performance of T-SPOT.*TB* on PEMCs and PBMCs. Patients who were clinically indeterminate were not included in the analysis of diagnostic value. Mean and standard deviation (SD) was used for data of normal distribution, while median and interquartile range (IQR) was used for data that were not normally distributed. Means and medians were compared using student’s t-test or Wilcoxon test as appropriate. Proportions were compared using Pearson’s Chi-square test. Significance was inferred for *P*<0.05 and statistical analysis was performed by SPSS16.0 (SPSS Inc, Chicago, IL, USA).

## Results

**Fig 1:**
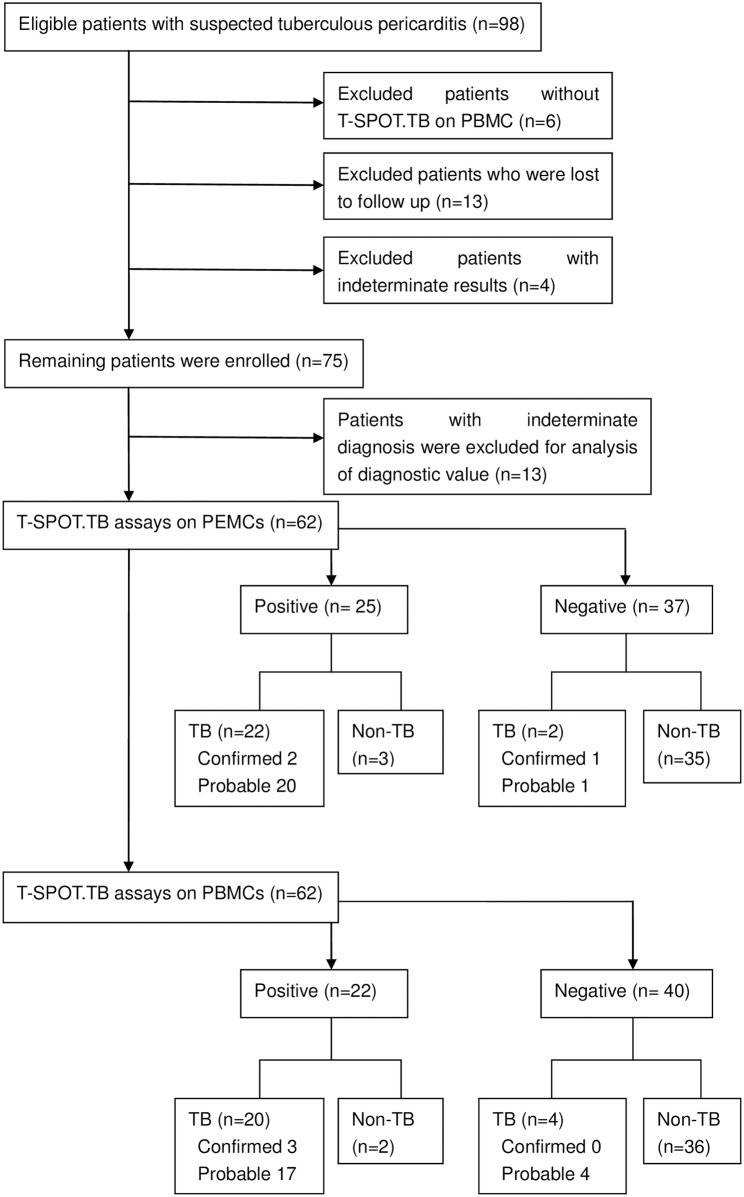
Flowchart of enrolling patients.

Among 98 patients with suspected tuberculous pericardial effusion, six patients who didn’t have T-SPOT.*TB* test on PBMCs, four patients with indeterminate T-SPOT.*TB* results, and 13 patients who were lost to follow up were excluded. None of the patients had uncertain result of T-SPOT.*TB*. 75 patients were enrolled (The flowchart is shown in Fig 1).

Among the 75 patients, 24 (32%) were diagnosed with tuberculous pericarditis, including three confirmed *TB* cases (Two with positive *MTB* culture in pericardial fluid(2/24, 8.3%), and one with positive *MTB* culture in sputum(1/8, 12.5%)in the presence of clinical and radiologic evidence of *TB* and in the absence of any other obvious cause associated with pericardial effusions) and 21 highly probable *TB* cases. At our last follow-up, some patients were still on therapy, and at this time, patients received a median time of 7.5 months (IQR 6–14) of anti-*TB*treatment.38 (51%) were diagnosed with nontuberculous pericarditis, including solid and hematological malignant tumors in 18 patients (47%), hematological diseases in two patients (5%, plasma cell dyscrasia and amyloidosis in one each patient), connective tissue diseases in five patients (13%), abnormal structure of thoracic duct leading to chylaceous pericardial effusion in six patients (16%), valvular heart disease in three patients (8%) and others in four patients (10%). 13 patients (17%)were clinically indeterminate. The basic characteristics are shown in Table 2.

**Table 2:** Baseline clinical characteristics and laboratory tests in 75 patients with suspected tuberculous pericarditis.

Characteristics	Tuberculous pericarditis (n = 24)	Non-tuberculous pericarditis (n = 38)	Clinically indeterminate (n = 13)
**Age—year(mean±SD)**	57±14	42±15	49±17
**Male—n (%)**	12(50)	17(45)	7(54)
**Symptoms—n (%)**			
** Breathlessness**	18 (60)	30 (79)	8 (62)
** Cough**	6 (25)	5 (13)	2 (15)
** Tachycardia**	4 (17)	1 (3)	1 (8)
** Edema**	4 (17)	12 (32)	8 (62)
** Fever**	3 (13)	3 (8)	1 (8)
** Chest pain**	2 (8)	2 (5)	0 (0)
**Pericardial tamponade—n (%)**	1 (4)	7 (18)	3 (23)
**Underlying conditions—n (%)**			
** HIV infection**	0 (0)	0 (0)	0 (0)
** Type 2** **diabetes****mellitus**	4 (17)	4 (11)	1 (8)
** Rheumatic diseases**	2 (8)	0 (0)	1 (8)
** Malignant tumor**	0 (0)	0 (0)	1 (8)
** ****Myelofibrosis**	1 (4)	0 (0)	0 (0)
** ****Post-renal****transplantation**	1 (4)	0 (0)	0 (0)
** Chronic kidney disease**	1 (4)	1 (3)	0 (0)
**Other sites involved of *TB*—n (%)**			
** Lung**	3 (12.5)	/	/
** Pleural**	2 (8)	/	/
**Laboratory tests**			
** Blood—n (%)**	24 (100)	38 (100)	13 (100)
** Leukocyte (Median, IQR)**	5.74(4.30~7.49)	6.96(4.91~9.32)	6.37(4.55~7.57)
Lymphocyte(*10^**9**^**/L,Median, IQR)**	1.37(0.78~1.75)	1.2(0.92~1.80)	1.04(0.87~1.21)
** ESR—n (%)**	24 (100)	36 (95)	13 (100)
** (mm/h, median, IQR)**	30 (10~47)	15 (9~41)	25 (6~40)
** hsCRP—n (%)**	21 (88)	36 (95)	12 (92)
** (mg/L, median, IQR)**	15.49 (4.06~46.27)	7.38(2.17~45.53)	18.58 (4.51~36.99)
**Pericardial effusion—n**	n = 24	n = 38	n = 13
** Appearance—n (%)**			
** Bloody**	8 (33)	20 (53)	6 (46)
** Yellow and** **muddy**	13 (54)	11 (29)	6 (46)
** Yellow and clear**	3 (13)	7 (18)	1 (8)
** Leukocyte—n (%)**	13 (54)	16 (42)	8 (62)
(*10^**6**^**/L, median, IQR)**	530 (82~2034)	190 (99~785)	383 (119~742)
** Monocyte (%, median, IQR)**	88(78~96)	82 (53~92)	62 (30~97)
** Adenosine deaminase—n (%)**	20 (83)	24 (63)	8 (62)
** (U/L, median, IQR)**	7.8 (5.6~31)	8.8 (4.8~15.3)	11.9 (6.6~29.4)

HIV: Human immunodeficiency virus; ESR: Erythrocyte sedimentation rate, normal range <20mm/h; hsCRP: Hypersensitive C reactive protein, normal range < 3mg/L.

### Sensitivity, specificity, predictive value and likelihood ratio of T-SPOT.*TB* on PEMCs and PBMCs

Among 24 patients with tuberculous pericarditis, T-SPOT.*TB* on PEMCs was positive in 22 patients, with a sensitivity of92%.T-SPOT.*TB* on PBMCs was positive in 20 patients, and the sensitivity of T-SPOT.*TB* on PBMCs was 83%.

Among 38 patients with nontuberculous pericarditis, T-SPOT.*TB* on PEMCs was negative in 35 patients, with a specificity of 92%. T-SPOT.*TB* on PBMCs was negative in 36 patients, with a specificity of 95%. T-SPOT.*TB* on PEMCs was positive in two patients with malignant tumors who had bloody pericardial effusion and one patient with connective tissue disease. T-SPOT.*TB* on PBMCs was positive in two patients without *TB* (Table 3). When combining T-SPOT.*TB* on PEMCs with PBMCs by parallel and serial testing algorithms[8], parallel test increased sensitivity to 100% but decreased specificity to 89%, while serial test increased specificity to 97% but decreased sensitivity to 75% (Table 3).

**Table 3:** Sensitivity, specificity, PPV, NPV, LR+, LR-, and area under the receiver operating characteristic curve (AUC) of T-SPOT.TB on PEMCs and PBMCs of patients with tuberculous pericarditis.

T-SPOT.*TB* on	Sensitivity(95%CI)	Specificity(95%CI)	PPV(95%CI)	NPV(95%CI)	LR+(95%CI)	LR-(95%CI)	AUC
**PEMCs**	92%(0.72–0.99)	92%(0.78–0.98)	88%(0.68–0.97)	95%(0.80–0.99)	11.61(3.89–34.63)	0.09(0.02–0.34)	0.942(0.874–1.01)
**PBMCs**	83%(0.62–0.95)	95%(0.81–0.99)	91%(0.69–0.98)	90%(0.75–0.97)	15.83(4.06–61.74)	0.18(0.07–0.43)	0.929(0.857–1.00)
**PBMCs&PEMCs (parallel)**	100%(0.83–1.00)	89%(0.74–0.97)	86%(0.66–0.95)	100%(0.87–1.00)	9.5(3.76–24.00)	0	/
**PBMCs&PEMCs (serial)**	75%(0.53–0.89)	97%(0.85–1.00)	95%(0.72–1.00)	86%(0.71–0.94)	28.5(4.06–199.87)	0.26(0.13–0.51)	/

Among the 25 patients with positive T-SPOT.*TB* on PEMCs, 22 were diagnosed with tuberculous pericarditis, and PPV was 88%; among 22 patients with positive T-SPOT.*TB* on PBMCs, 20 patients were diagnosed with tuberculous pericarditis, with a PPV of 91%.

Among the 37 patients with negative T-SPOT.*TB* on PEMCs, 35 were diagnosed with non tuberculous pericarditis, and NPV was 95%; among 40 patients with negative T-SPOT.*TB* on PBMCs, 36 were excluded of tuberculous pericarditis, and NPV was 90% (Table 3).

Additionally, the sensitivity and specificity of ESAT-6 on PEMCs was 92% and 92%, respectively. The sensitivity and specificity of CFP-10 on PEMCs was 75% and 97%, respectively.

### Comparison of frequencies of RD1 antigen-specific IFN-γ secreting T cells on PEMCs and PBMCs in patients with tuberculous pericarditis

In patients with tuberculous pericarditis, frequencies of SFCs of T-SPOT.*TB* on PEMCs appeared higher than that on PBMCs, however, the difference was not statistically significant (*P* = 0.183). Frequencies of SFCs of T-SPOT.*TB*,ESAT-6, and CFP-10 on PEMCs and PBMCs in patients with tuberculous pericarditis are shown in Table 4. Information of patients included is shown in S1 Data.

**Table 4:** Frequencies of T-SPOT.TB on PEMCs and PBMCs in patients with tuberculous pericarditis.

Frequencies (SFCs/10^6^MCs, median, IQR)	T-SPOT.*TB*	ESAT-6	CFP-10
**PEMCs**	172 (39~486)	62 (32~398)	72 (6~182)
**PBMCs**	66 (24~526)	52 (0~247)	24 (0~108)
***P* value**	0.183	0.225	0.166

## Discussion

Reports on the diagnostic value of T-SPOT.*TB* on pericardial effusion are limited. In this study, we found that the sensitivity, specificity, PPV and NPV of T-SPOT.*TB* on pericardial effusion for the diagnosis of tuberculous pericarditis is good.

Reasons of pericardial effusion may vary from different areas. *TB* is the dominant cause in developing countries (60%), where *TB* is endemic[9]. The proportion of a tuberculous origin of pericarditis is estimated to be at 4% in the Western world and up to 60% in the Republic of South Africa[10]. In our study, 32% patients had tuberculous pericardial effusion, and a study about causes of moderate to large pericardial effusion requiring pericardiocentesis in 140 Han Chinese patients showed that 40 cases (28.6%) had tuberculous pericardial effusion [11], which was similar to our results.

There have been a few case reports that explored the diagnostic value of T-SPOT.*TB* on pericardial effusion for the diagnosis of tuberculous pericarditis[10, 12, 13]. Results in our study suggested a high sensitivity (92%) and specificity (92%) of T-SPOT.*TB* on pericardial effusion. Previously, a study of 35 patients (12 with tuberculous pericardial effusion and 23 with nontuberculous pericardial effusion) showed that the sensitivity and specificity were 92% and 87% by ESAT-6 and 75% and 100% by CFP-10, using a cutoff value of 39SFCs/2.5*10^5^ for ESAT-6 and 92 for CFP-10[14], which was consistent to our results. Our study showed that the sensitivity of T-SPOT.*TB* on pericardial effusion was higher than that of peripheral blood, as previously reported, this might be due to the possible recruitment of *MTB*-specific effector T-lymphocytes at the site of infection. This phenomenon has been recently reported with a strong correlation with microbiologically proven pleural *TB*[15].

Among patients with tuberculous pericardial effusion, two had negative T-SPOT.*TB* on pericardial effusion and four had negative T-SPOT.*TB* on peripheral blood. Among these patients, two patients had low lymphocytes, another patient had a relatively low count of leukocyte in the pericardial effusion (0-5/HP), which might be the reason of the false negative results. And this was also reported by a previous study that the sensitivity of T-SPOT.*TB* would decrease with a lower lymphocyte count [16]. In another study which evaluated the diagnostic performance of T-SPOT.*TB* for extrapulmonary *tuberculosis* (E-*TB*) according to the site of infection[4],false-negative results were less common in samples from patients with chronic than with acute forms of E-*TB*.

Three patients with non-*TB* had positive T-SPOT.*TB* on pericardial effusion, of whom two had malignant tumors with pericardium metastasis which was confirmed by histopathology with bloody pericardial effusion. One patient had connective tissue disease (Systemic lupus erythematosus, SLE). Another study also showed that patients with malignant tumors and autoimmune diseases had false positive results in serous effusion, which indicated that aberrant immune activation might influence the diagnostic accuracy[5]. A previous cohort study of 631 patients with rheumatic diseases showed that patients with indeterminate results were more likely with SLE using a tumor necrosis factor (TNF)-α inhibitor[17]. Also, concomitant treatment with disease modifying anti-rheumatic drugs (DMARDs) has been documented as an essential driver of indeterminate results[18]. Further studies may be performed to have a better understanding of this.

Two patients with non-*TB* had positive T-SPOT.*TB* on peripheral blood. As patients with LTBI might have positive IGRA results, and an epidemiological investigation[19]showed that QuantiFERON (QFT) positivity rates ranged from 13% to 20% in rural China, indicating high prevalence of LTBI, which might explain the positive results in non-*TB* patients.

We further evaluated the diagnostic valued of combination of T-SPOT.*TB* on PEMCs with PBMCs by parallel and serial testing algorithms[8]. Sensitivity would be improved with parallel test and specificity would be improved with serial test. When patients had positive T-SPOT.*TB* on both PBMCs and PEMCs, sensitivity of T-SPOT.*TB* for diagnosis of tuberculous pericarditis is high, and if patients had negative T-SPOT.*TB* on both PBMCs and PEMCs, specificity of T-SPOT.*TB* for diagnosis of tuberculous pericarditis is high.

Currently, routine biochemistry studies in the pericardial fluid cannot differentiate tuberculous and nontuberculous pericarditis [11]. The yield of direct smear examination of pericardial fluid ranges from zero to 42%. Conventional culture of tubercle bacilli from pericardial fluid was positive in 53% of cases, and the diagnostic sensitivity of pericardial biopsy ranges from 10% to 64%. The sensitivity of PCR was higher with tissue specimens (80%) than with fluid specimens (15%), but is prone to contamination and false positivity [2]. As all these diagnostic tools are not ideal, T-SPOT.*TB* appears a relatively rapid diagnostic method with a good sensitivity and specificity.

### Limitations

We had a small number of microbiologically confirmed *TB* cases, so the sensitivity and specificity might be underestimated or overestimated. However, the prevalence of tuberculous pericarditis (confirmed and highly probable) in our study was similar to the results of other studies in our country and other developing countries where *TB* is endemic, therefore the diagnosis was relatively reliable. As data about diagnostic value of T-SPOT.*TB* on PEMCs was limited, our study provided some valuable information. In addition, this is a retrospective study in a single center, as our institution is the national diagnostic and treating centre for severe and complex diseases, patients included are always severe and complex, some bias may exist, and our finding may not be representative for other places.

In summary, we reported the diagnostic value of T-SPOT.*TB* on pericardial effusion for the diagnosis of tuberculous pericardial effusion in *TB* endemic area that was rarely reported before. It appears a valuable tool with high sensitivity and specificity, and diagnostic efficiency would be improved with the combined results of T-SPOT.*TB* on PBMCs and PEMCs.

## Supporting Information

S1 Data Information of the patients included. (DOCX)
