# Supplementary material for: Diagnostic Value of Interferon-γ Release Assays on Pericardial Effusion for Diagnosis of Tuberculous Pericarditis
Source: PLoS One. 2016 Oct 18;11(10):e0165008. doi: 10.1371/journal.pone.0165008 (PMC5068772; doi:10.1371/journal.pone.0165008)
Supplement: S1 Data — (DOCX) [file pone.0165008.s001.docx]

Data set

|  |  |  |  |  | On PEMCs | | | On PBMCs | | |
| --- | --- | --- | --- | --- | --- | --- | --- | --- | --- | --- |
| Case | Gender | Age(year) | Diagnosis | Other sites of TB | T-SPOT.TB | ESAT-6 | CFP-10 | T-SPOT.TB | ESAT-6 | CFP-10 |
| 1 | F | 75 | Confirmed TB(Positive MTB culture in pericardial fluid) | Lung | 12 | 4 | 8 | 3356 | 1320 | 2036 |
| 2 | M | 58 | Confirmed TB(Positive MTB culture in pericardial fluid) | Pleural | 2092 | 1600 | 492 | 656 | 548 | 108 |
| 3 | F | 70 | Confirmed TB(Positive MTB culture in sputum) | Lung | 3892 | 1960 | 1932 | 364 | 256 | 108 |
| 4 | M | 40 | Probable TB |  | 1456 | 832 | 624 | 1084 | 564 | 520 |
| 5 | F | 57 | Probable TB |  | 24 | 24 | 0 | 24 | 24 | 0 |
| 6 | F | 58 | Probable TB |  | 420 | 320 | 100 | 296 | 188 | 108 |
| 7 | F | 58 | Probable TB | Lung | 144 | 32 | 112 | 580 | 220 | 360 |
| 8 | M | 64 | Probable TB |  | 0 | 0 | 0 | 64 | 64 | 0 |
| 9 | F | 43 | Probable TB |  | 1680 | 1000 | 680 | 56 | 56 | 0 |
| 10 | F | 71 | Probable TB |  | 48 | 24 | 24 | 24 | 0 | 24 |
| 11 | M | 64 | Probable TB |  | 24 | 24 | 0 | 24 | 0 | 24 |
| 12 | F | 59 | Probable TB |  | 420 | 236 | 184 | 8 | 8 | 0 |
| 13 | F | 60 | Probable TB |  | 72 | 32 | 40 | 80 | 48 | 32 |
| 14 | M | 65 | Probable TB |  | 240 | 64 | 176 | 76 | 48 | 28 |
| 15 | F | 41 | Probable TB |  | 100 | 60 | 40 | 24 | 0 | 24 |
| 16 | M | 63 | Probable TB |  | 32 | 32 | 0 | 48 | 24 | 24 |
| 17 | M | 74 | Probable TB |  | 3764 | 1108 | 2656 | 4 | 0 | 4 |
| 18 | F | 39 | Probable TB |  | 200 | 140 | 60 | 4 | 4 | 0 |
| 19 | M | 57 | Probable TB |  | 272 | 104 | 168 | 32 | 0 | 32 |
| 20 | M | 81 | Probable TB | Pleural | 508 | 424 | 84 | 68 | 68 | 0 |
| 21 | F | 60 | Probable TB |  | 36 | 36 | 0 | 884 | 632 | 252 |
| 22 | M | 62 | Probable TB |  | 416 | 260 | 156 | 1160 | 580 | 580 |
| 23 | M | 41 | Probable TB |  | 60 | 36 | 24 | 0 | 0 | 0 |
| 24 | M | 19 | Probable TB |  | 88 | 48 | 40 | 72 | 72 | 0 |
| 25 | F | 68 | Non-TB |  | 4 | 0 | 4 | 0 | 0 | 0 |
| 26 | F | 57 | Non-TB |  | 0 | 0 | 0 | 4 | 4 | 0 |
| 27 | M | 49 | Non-TB |  | 20 | 8 | 12 | 0 | 0 | 0 |
| 28 | F | 49 | Non-TB |  | 8 | 4 | 4 | 0 | 0 | 0 |
| 29 | F | 46 | Non-TB |  | 0 | 0 | 0 | 0 | 0 | 0 |
| 30 | M | 68 | Non-TB |  | 0 | 0 | 0 | 4 | 0 | 4 |
| 31 | F | 62 | Non-TB |  | 40 | 40 | 0 | 4 | 4 | 0 |
| 32 | M | 39 | Non-TB |  | 0 | 0 | 0 | 0 | 0 | 0 |
| 33 | M | 32 | Non-TB |  | 20 | 12 | 8 | 0 | 0 | 0 |
| 34 | F | 39 | Non-TB |  | 4 | 4 | 0 | 0 | 0 | 0 |
| 35 | M | 61 | Non-TB |  | 20 | 12 | 8 | 0 | 0 | 0 |
| 36 | M | 47 | Non-TB |  | 0 | 0 | 0 | 0 | 0 | 0 |
| 37 | M | 34 | Non-TB |  | 0 | 0 | 0 | 0 | 0 | 0 |
| 38 | M | 56 | Non-TB |  | 8 | 0 | 8 | 0 | 0 | 0 |
| 39 | M | 30 | Non-TB |  | 0 | 0 | 0 | 4 | 4 | 0 |
| 40 | F | 56 | Non-TB |  | 0 | 0 | 0 | 0 | 0 | 0 |
| 41 | F | 24 | Non-TB |  | 4 | 0 | 4 | 0 | 0 | 0 |
| 42 | M | 14 | Non-TB |  | 0 | 0 | 0 | 0 | 0 | 0 |
| 43 | F | 63 | Non-TB |  | 0 | 0 | 0 | 0 | 0 | 0 |
| 44 | F | 42 | Non-TB |  | 52 | 28 | 24 | 0 | 0 | 0 |
| 45 | M | 59 | Non-TB |  | 0 | 0 | 0 | 8 | 4 | 4 |
| 46 | F | 54 | Non-TB |  | 12 | 4 | 8 | 16 | 0 | 16 |
| 47 | F | 24 | Non-TB |  | 0 | 0 | 0 | 0 | 0 | 0 |
| 48 | M | 62 | Non-TB |  | 68 | 68 | 0 | 64 | 64 | 0 |
| 49 | F | 40 | Non-TB |  | 4 | 0 | 4 | 4 | 4 | 0 |
| 50 | F | 18 | Non-TB |  | 0 | 0 | 0 | 4 | 0 | 4 |
| 51 | F | 48 | Non-TB |  | 0 | 0 | 0 | 0 | 0 | 0 |
| 52 | F | 25 | Non-TB |  | 8 | 4 | 4 | 0 | 0 | 0 |
| 53 | M | 28 | Non-TB |  | 0 | 0 | 0 | 0 | 0 | 0 |
| 54 | F | 17 | Non-TB |  | 20 | 12 | 8 | 8 | 0 | 8 |
| 55 | M | 44 | Non-TB |  | 4 | 4 | 0 | 0 | 0 | 0 |
| 56 | F | 44 | Non-TB |  | 16 | 4 | 12 | 0 | 0 | 0 |
| 57 | M | 22 | Non-TB |  | 16 | 12 | 4 | 4 | 4 | 0 |
| 58 | F | 35 | Non-TB |  | 8 | 0 | 8 | 4 | 0 | 4 |
| 59 | M | 45 | Non-TB |  | 0 | 0 | 0 | 8 | 8 | 0 |
| 60 | F | 49 | Non-TB |  | 0 | 0 | 0 | 12 | 8 | 4 |
| 61 | F | 20 | Non-TB |  | 0 | 0 | 0 | 24 | 24 | 0 |
| 62 | M | 36 | Non-TB |  | 0 | 0 | 0 | 4 | 0 | 4 |
| 63 | F | 36 | Clinically indeterminate |  |  |  |  |  |  |  |
| 64 | F | 77 | Clinically indeterminate |  |  |  |  |  |  |  |
| 65 | F | 65 | Clinically indeterminate |  |  |  |  |  |  |  |
| 66 | M | 45 | Clinically indeterminate |  |  |  |  |  |  |  |
| 67 | M | 23 | Clinically indeterminate |  |  |  |  |  |  |  |
| 68 | M | 34 | Clinically indeterminate |  |  |  |  |  |  |  |
| 69 | M | 62 | Clinically indeterminate |  |  |  |  |  |  |  |
| 70 | F | 51 | Clinically indeterminate |  |  |  |  |  |  |  |
| 71 | F | 35 | Clinically indeterminate |  |  |  |  |  |  |  |
| 72 | M | 60 | Clinically indeterminate |  |  |  |  |  |  |  |
| 73 | F | 64 | Clinically indeterminate |  |  |  |  |  |  |  |
| 74 | M | 58 | Clinically indeterminate |  |  |  |  |  |  |  |
| 75 | M | 24 | Clinically indeterminate |  |  |  |  |  |  |  |
